# Economic Analysis of Increasing Foot-and-Mouth Disease Vaccination Frequency: The Case of the Biannual Mass Vaccination Strategy

**DOI:** 10.3389/fvets.2020.557190

**Published:** 2020-10-16

**Authors:** Nursen Ozturk, Omur Kocak, Bouda Vosough Ahmadi

**Affiliations:** ^1^Department of Animal Breeding and Husbandry, Faculty of Veterinary Medicine, Istanbul University-Cerrahpasa, Istanbul, Turkey; ^2^European Commission for the Control of Foot and Mouth Disease, Food and Agriculture Organization of the United Nations, Rome, Italy

**Keywords:** FMD (Foot and Mouth Disease), vaccine, partial budgeting, Turkey, cattle

## Abstract

Biannual mass vaccination is a routinely applied foot-and-mouth disease (FMD) control strategy in Turkey. However, because biannual mass vaccination may leave significant immunity gaps, this strategy may cause economic losses because of possible FMD infections. In high-risk areas—such as border cities, it was suggested by the government to increase the vaccination intervals in order to decrease the FMD infection risk. This study analyses and compares the economic effects of a biannual mass vaccination regime and vaccination every 4 months as an alternative strategy in border cities by using partial budgeting approach. Biannual mass vaccination was used as a baseline scenario. Data on the impact of FMD on animal health and production parameters for 2018 were obtained from the OIE-WAHIS system and complemented by literature data and expert opinion. In the partial budgeting model, weight loss was considered as a major loss of income because majority of the farming systems are based on cattle fattening in the border cities of Turkey. Results revealed that the net economic impact, which is the benefit that exceeds the losses and costs of increasing the frequency of vaccination, is 76.4 TL ($15.9) per cattle. The sensitivity analysis showed that average body weight and weight losses when infected had more effect on net impact changes than market prices. The lower and upper FMD incidence variability resulted in 19.2 TL ($4) and 190.8 TL ($39.6) of net impact per cattle, respectively. The new FMD control strategy would make a total net economic impact of 5,274,836 TL ($1,094,250) for a population of 800,970 fattening cattle in border cities. The results of this study indicated that intense FMD control strategies may be more cost effective than the current control strategies, especially in high-risk areas. Future studies with more comprehensive epidemiological and economic data must be conducted to analyze and compare alternative FMD control strategies in Turkey.

## Introduction

Foot-and-mouth disease (FMD) imposes substantial production losses to farmers by causing decrease in milk and meat yield, fertility disorders, and mortality for young stock in cloven-hoofed animals (including cattle, pig, sheep, goat, and deer). It directly affects the production of animal origin food and eventually affects the product prices at a national scale in case of a large outbreak (1). Studies reported that the disease cause an 80% reduction in milk yield in its chronic form (2, 3), 2–5% of death among young stock (4), 25% decrease in live weight, and 10% increase in abortion rate (5), which, in total, results in 7–12% decrease in income of a farmer (6).

**Figure 1:**
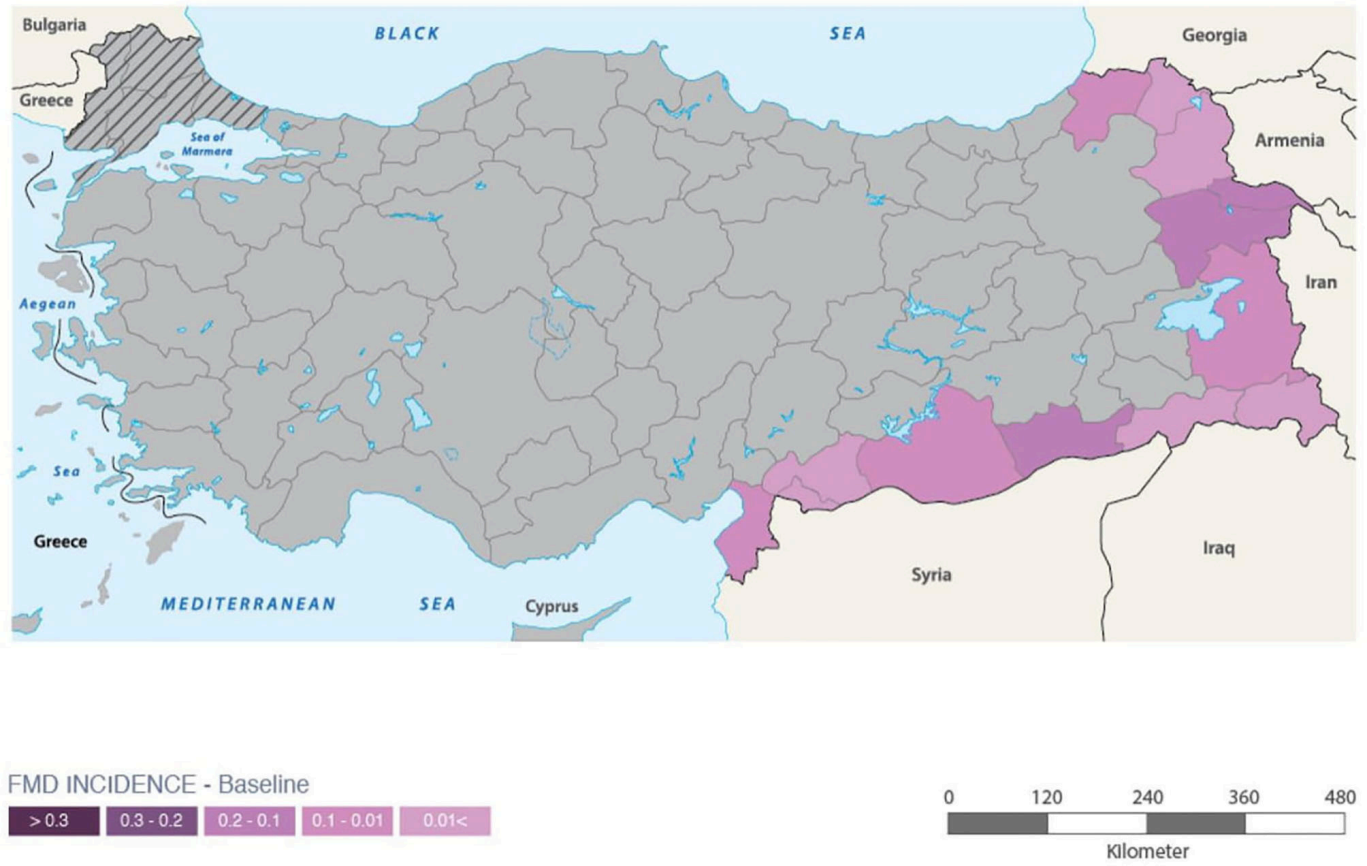
Map of Turkey. FMD incidences in border regions were highlighted relatively. The hashed lines show the Thrace region which is FMD free where the gray part depicts Anatolia.

Turkey is an FMD endemic country where the disease has been eradicated from the Thrace region but is still present in the Anatolian part of the country (Figure 1). The prevalence of the disease has been reduced from 45 to 5% between 2008 and year 2018 as a result of the government's control policies (7). The Turkish government aims to achieve an OIE status of FMD free with vaccination by 2023 by improving clinical surveillance programs in provinces along the border, vaccine effectiveness, and management of animal movement (8). Indeed, enhancing border security is a paramount strategy since a great number of studies demonstrated the role of legal and illegal animal movement in spreading FMD (9, 10). When comparing the border regions of Turkey with the West of Anatolia, a significant difference is observed in the number of outbreaks (11). This could be due to having FMD endemic neighbors, large-scale illegal cross-border movement, and insufficient biosecurity. In high-risk areas, there is an increasing need to sustain a high level of vaccine efficacy and protection in order to ensure FMD control throughout the country. Therefore, in regions where the outbreak incidence is high, it is recommended to increase the vaccination intervals by the government.

Cattle breeding in border cities of Turkey is conducted by smallholders and farmers raising local breeds, indigenous breeds, and their crosses for milk and meat production. Cross breeds of Holsteins, Simmental, and Brown Swiss are the most favored breeds in these areas. Male calves that are born on dairy farms are taken at the age of 4–8 months and at about 120–200 kg of live weight for fattening purposes and mostly for the Kurban festival of slaughter. These animals are then sold at a weight of 300–600 kg depending on the breed after they become 2 years old. Calves receive their first FMD vaccine (containing six PD50) doses at the age of 2 months. Vaccination is applied biannually as the current strategy of the government to control and reduce FMD outbreaks.

The time since the last vaccination have an effect on expected immunity. With biannual mass vaccination, sustained immunity level cannot be achieved due to rapidly declining antibody titres, which require multiple doses of vaccination (12). A high-potency vaccine or a vaccination scheme with increased frequency of administration is required in order to sustain immunity level. However, engaging farmers in disease control strategies is quite challenging, as smallholders' willingness to participate in a disease control program is reported to be low if the vaccination is not free of charge (13). Economic impact assessment studies of disease control policies using analytical approaches such as partial budgeting would encourage farmers to participate in the disease control program by demonstrating the obtained benefit from alternative policies. Furthermore, economic impact assessment studies support the veterinary authorities with decision support information to justify and adjust control plans if necessary.

Partial budgeting is an analytical economic method that can be used for comparing alternative disease control measures on a farm. It is used to estimate possible changes that are caused by a proposed disease control plan by considering benefits and costs that are available (14). To our knowledge in Turkey, FMD control measures have not been compared by using any economic modeling techniques before. In other FMD endemic settings, studies using partial budgeting approach determined a positive net return considering the application of mass vaccination campaigns (15, 16).

In this study, we aim to analyze and compare the net economic impact of increasing the vaccination frequency to 4 months in border districts of Turkey vs. the current biannual vaccination policy, using partial budget analysis. This analysis provides supportive information for the policy makers in order to protect smallholder income for a sustainable production and prevent losses, which are caused by FMD.

## Materials and Methods

### Data

Primary epidemiological data was obtained through the OIE's 2018 country reports for each city in border districts. Financial information was obtained through the market values for the year 2018. Literature values and expert views were also included as secondary data. FMD incidences and mortality for each city in 2018 are given in Appendix 1. We hypothesized that increasing vaccination frequency would decrease the FMD incidence, mortality, and morbidity. Therefore, in order to reflect the effect of increasing vaccination frequency on FMD incidence, mortality, and morbidity, we multiplied the observed FMD incidences, mortality, and morbidity by the relative risk (RR) values considering the number of received doses of FMD vaccination, which was reported by Knight–Jones et al. (17).

The formulas for estimating the FMD incidence, mortality, and morbidity used in the scenario are presented below:

(1) $$\begin{array}{r} \text{In}{\text{c}}_{\text{Sc}}:\text{In}{\text{c}}_{\text{Bc}}\;*\text{ RR} \end{array}$$

with Inc_Sc_ being the FMD incidence rate for the cth city in the border district used in vaccination in the 4-months scenario. The Inc_Sc_ value is calculated by multiplying the baseline FMD incidence for the c^th^ city with the relative risk value.

(2) $$\begin{array}{r} \text{Mor}{\text{t}}_{\text{Sc}}:\text{Mor}{\text{t}}_{\text{Bc}}\;*\text{ RR} \end{array}$$

with Mort_Sc_ being the FMD mortality rate for the cth city in the border district used in the scenario. The Mort_Sc_ value is calculated by multiplying the baseline FMD mortality for the cth city with the relative risk value.

(3) $$\begin{array}{r} \text{Mor}{\text{b}}_{\text{S}}:\text{Mor}{\text{b}}_{\text{B}}\;*\text{ RR} \end{array}$$

with Morb_S_ being the FMD morbidity rate for the district level used in the scenario. The Morb_S_ value is calculated by multiplying the baseline FMD morbidity level with the relative risk value.

### Partial Budgeting Approach

In order to compare the impact of vaccination in a 4-month-interval strategy in border regions to the baseline scenario, we applied a deterministic economic model by using partial budget analysis. FMD incidence, morbidity rate, mortality rate, weight loss when infected, average body weight, average duration of illness, value of live weight, cost of replacement, cost of FMD treatment, cost of FMD vaccination, cost of feed, and cost of veterinary services were included as inputs. The input variables used in the partial budget analysis and sources are given in Table 1.

**Table 1:** Partial budget input variables and sources.

**Inputs**	**Baseline**	**Scenario**	**Source**
	**Value**	**Value**	
Foot-and-mouth disease (FMD) incidence^#^, %	12.4 ± 0.1^a^	5.9 ± 0.1^a^	Calculation (7)
Morbidity rate^#^, %	60.0 (42.2–72.3)^b^	30.0 (20.9–34.3)^b^	(18)
Mortality rate^#^, %	1.4 ± 2.1^a^	0.6 ± 0.9^a^	Calculation (7)
Weight loss when infected, %	25 (15–30)^b^	25 (15–30)^b^	(5)
Average body weight, kg	355^c^	355^c^	(5)
Average duration of illness, d	13.3 ± 5.5^a^	13.3 ± 5.5^a^	Expert survey
Value of live weight, TL	15.3 ± 1.3^a^	15.3 ± 1.3^a^	(19)
Cost of replacement, TL	6,673.9 ± 1,266.2^a^	6,673.9 ± 1,266.2^a^	(19)
Cost of FMD treatment, TL	441.7 ± 210.8^a^	441.7 ± 210.8^a^	Expert survey
Cost of FMD vaccination, TL	9.6 (0.4–3)^b^	14.4 (0.4–3)^b^	(12)
Cost of feed, TL/day	11.8 ± 3.8^a^	11.8 ± 3.8^a^	(20)
Cost of veterinary services, TL/day	0.8^c^	0.8^c^	Calculation (21)

# *When calculating the FMD incidence, morbidity, and mortality values for the scenario, the baseline incidence, morbidity, and mortality values were multiplied by the relative risk ratios. The relative risk ratio is the likelihood of an animal to become infected considering the received number of FMD vaccination doses (17)*.

*TL, Turkish Lira (Turkish currency)*.

a *Normal distribution: mean ± SD*.

b *Normal distribution: mean (CI 95%)*.

c *Data available with mean only*.

Weight loss was considered as the major production loss because in the eastern part of Turkey, fattening is an important production system when comparing border cities to other regions of the country. Therefore, other production parameters such as decrease in milk production or increase in abortion rate were not considered in the analysis.

In the partial budgeting model, we also included the potential immunity gap (IG), which would potentially be caused by a decrease in antibody levels after receiving an FMD vaccine dose. The antibody levels were reported to decline by 0.5% per day (17). We multiplied the reported daily antibody decrease for 6- and 4-months of vaccination intervals in order to find out immunity gaps for base (IG_B_) and scenario (IG_S_).

Our partial budget model consists of the components that are described below:

(1) Additional return (Ar): is primarily derived from the weight gain achieved by a healthy cattle, calculated as the average duration of illness multiplied by the estimated mean daily weight gain and the value of the live weight per kg. This value is then multiplied by the annual disease incidence, morbidity, and immunity gap.

(4) $$\begin{array}{r} \sum_{Sc}Ar=\left[\left(\text{AD}{\text{G}}_{\text{healthy}}\;*\text{ t}\right)-\left(\text{AD}{\text{G}}_{\text{healthy}}\;*\;{\text{W}}_{\text{loss}}\;*\text{ t}\right)\right]\;*\;{\text{P}}_{\text{LW}}\\ \;*\text{ In}{\text{c}}_{\text{Sc}}\;*\text{ Mor}{\text{b}}_{\text{S}}\;*\text{ I}{\text{G}}_{\text{S}} \end{array}$$

(5) $$\begin{array}{r} \sum_{Bc}Ar=\left[\left(\text{AD}{\text{G}}_{\text{healthy}}\;*\text{ t}\right)-\left(\text{AD}{\text{G}}_{\text{healthy}}\;*\;{\text{W}}_{\text{loss}}\;*\text{ t}\right)\right]\;*\;{\text{P}}_{\text{LW}}\;*\;\\ \text{ In}{\text{c}}_{\text{Bc}}\;*\text{ Mor}{\text{b}}_{\text{B}}\;*\text{ I}{\text{G}}_{\text{B}} \end{array}$$

where ADG_healthy_ represents the average daily weight gain of healthy cattle, t represents the average duration of the disease, P_LW_ stands for the value of live weight, and W_loss_ represents the percentage of weight loss when infected.

(2) Reduced cost (Rc): is primarily derived from the cost of disease treatment, cost of weight loss, and cost of replacement animals. The summation of these costs is multiplied by the disease incidence, morbidity, and immunity gap.

(6) $$\begin{array}{r} \sum_{\text{Sc}}\text{Rc}=\left[\text{Treat}.\text{cost}+{\text{W}}_{\text{loss}}.\text{cost}+\left(\text{Rep}.\text{cost }*\text{ Mor}{\text{t}}_{\text{Sc}}\right)\right]\\ \;*\text{ In}{\text{c}}_{\text{Sc}}\;*\text{ Mor}{\text{b}}_{\text{S}}\;*\text{ I}{\text{G}}_{\text{S}} \end{array}$$

(7) $$\begin{array}{r} \sum_{\text{Bc}}\text{Rc}=\left[\text{Treat}.\text{cost}+{\text{W}}_{\text{loss}}.\text{cost}+\left(\text{Rep}.\text{cost }*\text{Mor}{\text{t}}_{\text{Bc}}\right)\right]\\ \;*\text{ In}{\text{c}}_{\text{Bc}}\;*\text{ Mor}{\text{b}}_{\text{B}}\;*\text{ I}{\text{G}}_{\text{B}} \end{array}$$

(8) $$\begin{array}{r} {\text{W}}_{\text{loss}}.\text{cost}={\text{W}}_{\text{loss}}\;*\text{ A}{\text{v}}_{\text{LW}}\;*\;{\text{P}}_{\text{LW}} \end{array}$$

where Treat.cost represents the FMD treatment cost per infected cattle, W_loss_.cost is the cost of weight loss, and Rep.cost stands for the replacement cost in case of a death caused by FMD. Cost of weight loss is calculated by multiplying the average percentage of weight loss by the average body weight at the time of infection and price of live weight. Av_LW_ represents the average live weight of cattle at the time of infection.

(3) Return forgone (Rf), was considered to be zero because selling dead animals is not practiced in Turkey.

(4) Additional costs (Ac): These are of the alternative plan, referencing the purchase and administration of the FMD vaccine. Furthermore, due to lower mortality rates, additional feed and veterinary cost are included as extra costs.

(9) $$\begin{array}{r} \sum_{Sc}Ac=\left(\text{Vac}.\text{cost }*\;3\right)+\text{Add}.\text{feed}.\text{cost}+\text{Add}.\text{vet}.\text{cost} \end{array}$$

(10) $$\begin{array}{r} \sum_{Sc}Add.feed.cost=\text{Feed}.\text{cost }*\text{ In}{\text{c}}_{\text{Sc}}\;*\text{ Mor}{\text{b}}_{\text{S}}\;*\text{ Mor}{\text{t}}_{\text{Sc}}\;*\text{ I}{\text{G}}_{\text{S}} \end{array}$$

(11) $$\begin{array}{r} \sum_{Sc}Add.vet.cost=\text{Vet}.\text{cost }*\text{ In}{\text{c}}_{\text{Sc}}\;*\text{ Mor}{\text{b}}_{\text{S}}\;*\text{ Mor}{\text{t}}_{\text{Sc}}\;*\text{ I}{\text{G}}_{\text{S}} \end{array}$$

(12) $$\begin{array}{r} \sum_{Bc}Ac=\left(\text{Vac}.\text{cost }*\;2\right)+\text{Add}.\text{feed}.\text{cost}+\text{Add}.\text{vet}.\text{cost} \end{array}$$

(13) $$\begin{array}{r} \sum_{Bc}Add.feed.cost=\text{Feed}.\text{cost }*\text{ In}{\text{c}}_{\text{Bc}}\;*\text{ Mor}{\text{b}}_{\text{B}}\;*\text{ Mor}{\text{t}}_{\text{Bc}}\;*\text{ I}{\text{G}}_{\text{B}} \end{array}$$

(14) $$\begin{array}{r} \sum_{Bc}Add.vet.cost=\text{Vet}.\text{cost }*\text{ In}{\text{c}}_{\text{Bc}}\;*\text{ Mor}{\text{b}}_{\text{B}}\;*\text{ Mor}{\text{t}}_{\text{Bc}}\;*\text{ I}{\text{G}}_{\text{B}} \end{array}$$

where Vac.cost represents the FMD vaccination cost, Add.feed.cost stands for the additional feed cost due to lower mortality that will be needed for a cattle per year, and Add.vet.cost is the additional veterinary costs that caused a lower mortality that will be needed in treating healthier animals per year.

### Sensitivity Analysis

Due to the fluctuation of prices and disease parameters, we applied sensitivity analysis by considering the minimum, most likely, and maximum values of cattle price, value of live weight, FMD treatment cost, percentage of weight loss when infected, duration of disease, and average body weight at the time of infection. Furthermore, in order to understand how the lower and upper disease incidences affects the net impact of vaccination in 4 months, 50% of the lower and upper values of the observed FMD incidences were included in the sensitivity analysis.

## Results

**Figure 2:**
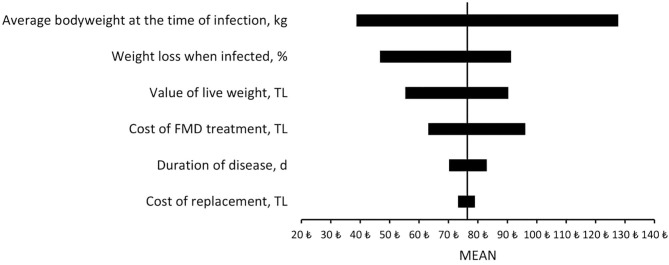
Sensitivity analysis results of FMD vaccination strategy.

The results of the partial budget analysis revealed that the net impact of increasing vaccination frequency by up to three times per year in high-risk areas would be 76.4 TL/cow. When the minimum and maximum values of the disease and economic parameters were included in the sensitivity analysis, the average body weight at the time of infection and weight loss when infected were found to be the most influencing parameters that affect the outcome of the partial budget model (Figure 2). When the minimum value for the percentage of weight loss was considered, the result of the partial budget analysis was 38.7 TL/cow, which was the lowest result. Furthermore, the maximum value of the average body weight at the time of infection resulted in the highest net impact of the overall partial budget analysis (127.7 TL/cow). In this study, the results of the sensitivity analysis showed a positive net impact for all included parameters, even for the most influencing parameters, and changing economic parameters did not affect the net impact of increasing the vaccination interval strategy (Table 2).

**Table 2:** Changes in partial budget analysis results by applying the minimum, most likely, and maximum input values.

**Variable**	**Value**	**Net impact of PB (TL)**	**Net impact of PB ($)**	**Change from baseline (TL)**	**Change from baseline ($)**
Cost of replacement, TL	6,673.9	76.4			
Minimum	4,003.7	73.2	15.2	3.2	0.7
Maximum	8,866.2	79.0	16.4	2.6	0.5
Value of live weight, TL	15.3	76.4			
Minimum	11.6	55.3	11.5	21.1	4.4
Maximum	17.7	90.4	18.7	13.9	2.9
Duration of disease, days	13.3	76.4			
Minimum	7	70.2	14.6	6.2	1.3
Maximum	20	83.0	17.2	6.6	1.4
Average body weight, kg	355	76.4			
Minimum	175	38.7	8.0	37.7	7.8
Maximum	600	127.7	26.5	51.3	10.6
Weight loss when infected, %	25	76.4			
Minimum	15	46.7	9.7	29.7	6.2
Maximum	30	91.3	18.9	14.9	3.1
Cost of FMD treatment, TL	441.7	76.4			
Minimum	200	63.1	13.1	13.2	2.8
Maximum	800	96.1	19.9	19.7	4.1

*TL, Turkish Lira (Turkish currency)*.

Varying the FMD incidence by 50% lower and upper values showed a positive net impact (Table 3). In one case of increasing the FMD incidence by 50%, the sensitivity analysis results were 190.8 TL/cow and 19.2 TL/cow when the incidence rate was lowered by 50%. This result implies that even with a low risk of FMD, increasing the vaccination interval was still profitable.

**Table 3:** Results of sensitivity analysis on FMD annual incidence while comparing baseline and partial budget (PB) results.

**Variable**	**Mean value**	**Net impact of PB (TL)**	**Net impact of PB ($)**
FMD incidence (baseline)	0.12	76.4	15.9
Lower incidence estimation	0.06	19.2	4.0
Upper incidence estimation	0.24	190.8	39.6

*TL, Turkish Lira (Turkish currency)*.

In Table 4, gain, losses, and net impact of the partial budget analysis were shown for each city in the bordering region. The total net impact was found to be 5,274,836 TL. Two cities located in the East Anatolia Region, Agri (3,026,633.6 TL) and Igdir (851,181.2 TL), were determined as the cities with the highest net impact. However, also in the same region, Van (1,684.5 TL) was the city with the lowest net impact.

**Table 4:** Gain (reduced costs and additional revenue), loss (extra cost and revenue forgone), and net impact from partial budget analysis of vaccination in the 4-months strategy compared with the baseline per each border city in 2018.

**City**	**Gain (TL)**	**Gain ($)**	**Cost (TL)**	**Cost ($)**	**Net impact (TL)**	**Net impact ($)**	**Cost/Benefit**
Agri	3,104,790.8	644,080.7	78,157.3	16,213.5	3,026,633.6	627,867.1	0.03
Ardahan	141,358.7	29,324.5	12,848.0	2,665.3	128,510.7	26,659.2	0.09
Artvin	1,015,849.6	210,735.3	305,117.9	63,295.9	710,731.7	147,439.4	0.30
Gaziantep	157,763.7	32,727.7	155,975.0	32,356.6	1,788.7	371.1	0.99
Hatay	424,401.9	88,041.1	292,581.2	60,695.2	131,820.6	27,345.8	0.69
Igdir	876,803.4	181,890.5	25,622.2	5,315.3	851,181.2	176,575.3	0.03
Kars	160,154.6	33,223.6	17,562.8	3,643.4	142,591.8	29,580.3	0.11
Mardin	511,387.5	106,086.0	494,366.0	102,554.9	17,021.5	3,531.1	0.97
Sanliurfa	286,488.6	59,431.3	23,616.8	4,899.3	262,871.8	54,532.1	0.08
Van	3,341.27	693.1	1,656.8	343.7	1,684.5	349.5	0.50
TOTAL	6,682,340.0	1,386,233.8	1,407,503.9	291,983.0	5,274,836.1	1,094,250.8	0.36

*TL, Turkish Lira (Turkish currency)*.

## Discussion

In this study, a deterministic approach was used to determine whether the proposed vaccination strategy is profitable for the farmers. The partial budget analysis showed that increasing the vaccination frequency produced a positive net impact of 76.4 TL/cow, indicating that FMD causes severe losses to farmers. In the analysis, the economic consequences of weight loss due to foot-and-mouth disease were the only effect that was considered, as the majority of farms in Eastern Turkey are for fattening. In a study conducted by Truong et al. (16), it was found that dairy farmers would get a higher benefit than beef farms by applying biannual FMD vaccination. Therefore, the net impact of partial budget analysis would possibly be higher if the effect of milk reduction and fertility disorder had been included.

A sensitivity analysis was performed using both disease and economic parameters including cattle value, value of live weight, duration of disease, average body weight at the time of infection, percentage of weight loss when infected, and cost of treatment. The net benefit of increasing vaccination interval under uncertainty remained at 38.7 TL/cow or higher.

Vaccination is an important strategy in controlling FMD. Although in Turkey preventive vaccination campaign is applied biannually in Anatolia and three times a year in the Thrace (FMD free) region, full protection cannot be achieved. If a booster dose is not applied during the vaccination campaigns, there can be an immunity gap within 6 months (12), which requires several doses of vaccine. This study supports the introduction of a new vaccination interval scheme.

The overall net impact of the proposed vaccination scenario in border regions shows that some cities are more likely to gain a higher net impact, possibly due to their higher FMD incidence rate. The outcome reveals that an effective disease control strategy will be economically beneficial especially in high-risk areas. For countries with a limited export opportunity, controlling diseases is recommended to target high-risk areas to generate a positive economic return (4). It was estimated that the net benefit of the proposed vaccination scenario for the city Agri is 1.7% of its gross domestic product from agriculture (22).

Most farms are smallholding in border cities in Turkey, and they are dependent on livestock. Although extensive farmers are less motivated to participate in FMD control programs (23), the farmers' willingness to pay for vaccines and their participation in vaccination campaigns are reported to be increased through awareness of vaccine benefits (24). In Turkey, FMD vaccination is given without any charge; farmers only pay for the vaccine application cost to the state veterinarian. However, besides the cost of vaccine, the farmers' participation in vaccination campaigns is reported to decrease by considering the side effects of vaccination such as abortion, decrease in milk production for a few days after vaccination, and animals becoming sick (25). This indicates a need to explain the side effects of vaccine to avoid mistrust and increase the uptake. In addition, presenting the results of economic studies showing the benefits of disease control programs are likely to motivate smallholders for their participation.

Although in this study a positive net economic impact of 76.4 TL/cow was revealed under the proposed vaccination scheme, there may be some debates about its feasibility due to limited human resources. In Turkey, FMD vaccine campaigns are applied by the state veterinary service. However, in regions where human sources and transportation availability are limited, vaccine application could also be done by private veterinarians besides state veterinarians. Furthermore, increasing the frequency of vaccination in order to close the immunity gap will require a higher number of FMD vaccine to be produced. Therefore, optimization studies focusing on vaccine production, storage, delivery, and accounting for changes in the FMD incidence rate are essential to further support our findings.

One limitation of this study was that epidemiological parameters for the vaccination scenario could not be obtained by conducting a field study. Hypothetically, current epidemiologic parameters for the year 2018 were multiplied by the relative risk ratio (17).

## Conclusion

The partial budget analysis revealed that increasing vaccination frequency would result in a positive net economic impact of 76.4 TL/cow for farmers. Therefore, controlling FMD outbreaks signifies a socioeconomic gain to farmers that could improve participation in disease prevention programs. This study provides additional information for policy makers in order to adjust their FMD control strategy in border cities, taking into account regional variation in infection rates. Further studies are recommended, focusing on alternative FMD control strategies by using more comprehensive epidemiological and financial data throughout the country.

## Data Availability Statement

The original contributions presented in the study are publicly available. This data can be found here: https://www.oie.int/.

## Ethics Statement

Ethical review and approval was not required for the animal study because related data was obtained through OIE-WAHIS system which is available to public use. This was stated in the manuscript.

## Author Contributions

NO and BV designed the study and reviewed the results. NO and OK contributed to data access. All authors has been read, approved, and contributed to analyses.

## Conflict of Interest

The authors declare that the research was conducted in the absence of any commercial or financial relationships that could be construed as a potential conflict of interest.
