# Supplementary material for: Economic Analysis of Increasing Foot-and-Mouth Disease Vaccination Frequency: The Case of the Biannual Mass Vaccination Strategy
Source: Front Vet Sci. 2020 Oct 16;7:557190. doi: 10.3389/fvets.2020.557190 (PMC7597382; doi:10.3389/fvets.2020.557190)
Supplement: Supplementary file 1 [file Table_1.DOCX]

Appendix 1. Reported disease parameters for border cities in Turkey by World Organization for Animal Health (OIE) in 2018

| **City** |  | **Susceptible** |  | **Cases** |  | **Deaths** |  | **Killed and disposed** |  | **Slaughtered** |  | **Incidence (%)** |  | **Mortality (%)** |
| --- | --- | --- | --- | --- | --- | --- | --- | --- | --- | --- | --- | --- | --- | --- |
| Agri |  | 235 |  | 50 |  | 0 |  | 0 |  | 0 |  | 21.3 |  | 0.0 |
| Ardahan |  | 1680 |  | 99 |  | 0 |  | 0 |  | 0 |  | 5.9 |  | 0.0 |
| Artvin |  | 67 |  | 22 |  | 2 |  | 0 |  | 0 |  | 32.8 |  | 3.0 |
| Gaziantep |  | 5764 |  | 274 |  | 86 |  | 4 |  | 1 |  | 4.8 |  | 1.6 |
| Hatay |  | 362 |  | 42 |  | 6 |  | 0 |  | 4 |  | 11.6 |  | 2.8 |
| Igdir |  | 682 |  | 125 |  | 0 |  | 0 |  | 0 |  | 18.3 |  | 0.0 |
| Kars |  | 1208 |  | 59 |  | 0 |  | 0 |  | 0 |  | 4.9 |  | 0.0 |
| Mardin |  | 47 |  | 8 |  | 3 |  | 0 |  | 0 |  | 17.0 |  | 6.4 |
| Sanliurfa |  | 354 |  | 23 |  | 0 |  | 0 |  | 0 |  | 6.5 |  | 0.0 |
| Van |  | 6573 |  | 71 |  | 0 |  | 0 |  | 0 |  | 1.1 |  | 0.0 |

Source: World Animal Health Information System (WAHIS), 2018 country report
